# Remodeling the endoplasmic reticulum proteostasis network restores proteostasis of pathogenic GABA_A_ receptors

**DOI:** 10.1371/journal.pone.0207948

**Published:** 2018-11-27

**Authors:** Yan-Lin Fu, Dong-Yun Han, Ya-Juan Wang, Xiao-Jing Di, Hai-Bo Yu, Ting-Wei Mu

**Affiliations:** 1 Department of Physiology and Biophysics, Case Western Reserve University School of Medicine, Cleveland, Ohio, United States of America; 2 Center for Proteomics and Bioinformatics and Department of Epidemiology and Biostatistics, Case Western Reserve University School of Medicine, Cleveland, Ohio, United States of America; 3 School of Chemistry and Molecular Bioscience & Molecular Horizons, University of Wollongong, Wollongong, Australia; University of Pittsburgh, UNITED STATES

## Abstract

Biogenesis of membrane proteins is controlled by the protein homeostasis (proteostasis) network. We have been focusing on protein quality control of γ-aminobutyric acid type A (GABA_A_) receptors, the major inhibitory neurotransmitter-gated ion channels in mammalian central nervous system. Proteostasis deficiency in GABA_A_ receptors causes loss of their surface expression and thus function on the plasma membrane, leading to epilepsy and other neurological diseases. One well-characterized example is the A322D mutation in the α1 subunit that causes its extensive misfolding and expedited degradation in the endoplasmic reticulum (ER), resulting in autosomal dominant juvenile myoclonic epilepsy. We aimed to correct misfolding of the α1(A322D) subunits in the ER as an approach to restore their functional surface expression. Here, we showed that application of BIX, a specific, potent ER resident HSP70 family protein BiP activator, significantly increases the surface expression of the mutant receptors in human HEK293T cells and neuronal SH-SY5Y cells. BIX attenuates the degradation of α1(A322D) and enhances their forward trafficking and function. Furthermore, because BiP is one major target of the two unfolded protein response (UPR) pathways: ATF6 and IRE1, we continued to demonstrate that modest activations of the ATF6 pathway and IRE1 pathway genetically enhance the plasma membrane trafficking of the α1(A322D) protein in HEK293T cells. Our results underlie the potential of regulating the ER proteostasis network to correct loss-of-function protein conformational diseases.

## Introduction

About 1/3 of the eukaryotic proteins, including all membrane proteins, enter the endoplasmic reticulum (ER) for their protein folding [1–3]. Many mutations in ion channel proteins result in their misfolding, and the mutant proteins are retained in the ER and degraded by the ER-associated degradation (ERAD) pathway [4–6]. Consequently, fewer ion channels reach their working destination. This leads to loss of their function and corresponding disease phenotypes [7]. Examples of such conformational diseases include cystic fibrosis resulting from cystic fibrosis transmembrane conductance regulator (CFTR) misfolding [8], type 2 long QT syndrome resulting from trafficking deficiency of human *ether*-*à*-*go*-*go*-related gene (hERG) channels [9,10], congenital myasthenic syndromes resulting from misfolding of nicotinic acetylcholine receptors [11], and idiopathic epilepsy resulting from misfolding of γ-aminobutyric acid type A (GABA_A_) receptors [12,13]. We concentrate on proteostasis maintenance of GABA_A_ receptors because this topic has been under studied.

**Fig 1:**
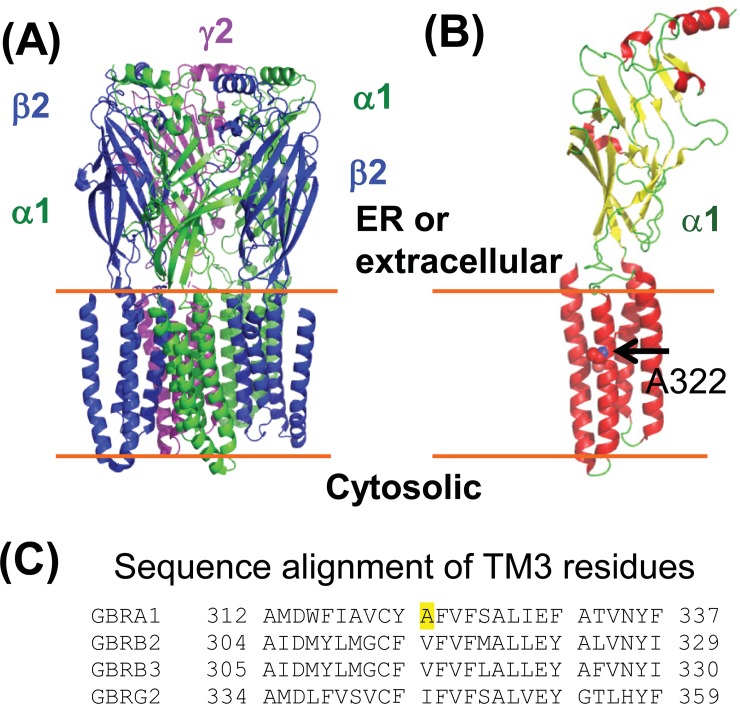
Molecular structures of GABA_A_ receptors. (**A**) A cartoon representation of the major pentameric GABA_A_ receptor subtype in the central nervous system. It contains two α1 subunits, two β2 subunits, and one γ2 subunit. This model was constructed from the cryo-EM structure (6D6U.pdb) [22] by using PYMOL. (**B**) Topology of the α1 subunit. The large N-terminal domain resides in the ER lumen or extracellular space. Ala322, displayed as a space-filling model and indicated by an arrow, is located in the third transmembrane (TM3) helix. (**C**) Sequence alignment of TM3 residues of the α1, β2, β3, and γ2 subunits of GABA_A_ receptors. The sequences are from the following Uniprot entries: GBRA1, P14867; GBRB2, P47870-1; GBRB3, P28472-1; GBRG2, P18507-2. The A322 residue in the α1 subunit is highlighted in yellow. Hydrophobic residues are conserved in this position.

GABA_A_ receptors are the primary inhibitory ligand-gated ion channels in mammalian central nervous system [14,15] and provide most of the inhibitory tone to balance the tendency of excitatory neural circuits to induce hyperexcitability, thus maintaining the excitatory-inhibitory balance [16]. Loss of function of GABA_A_ receptors causes idiopathic epilepsy and other neurological diseases [17–21]. GABA_A_ receptors are pentamers that are assembled from eight subunit classes: α1–6, β1–3, γ1–3, δ, ε, θ, π, and ρ1–3. The most common subtype in the brain is constructed from two α1 subunits, two β2 subunits, and one γ2 subunit (**Fig 1A**). Each of these subunits shares a common topology, containing a large extracellular (or ER luminal) N-terminal domain, four transmembrane (TM) helices (TM1-TM4), a major intracellular loop connecting TM3 and TM4, and a short extracellular (or ER luminal) C-terminus (**Fig 1B**) [22–24]. Individual subunits need to achieve their native structures in the ER [25,26] and assemble with other subunits properly to form a pentamer on the ER membrane [27,28] for subsequent trafficking to the plasma membrane. Due to their complex structures, the folding and assembly of GABA_A_ receptors are inefficient. Many mutations in individual subunits aggravate such an inefficient process.

Recent advances in genetics identified an increasing number of mutations in α1, β2, β3 and γ2 subunits of GABA_A_ receptors that are associated with idiopathic epilepsies [29–34]. Numerous disease-causing mutations in GABA_A_ receptor subunits cause their protein misfolding and reduce their assembly in the ER, leading to excessive ERAD [18,35]. One well-characterized ERAD substrate is the α1(A322D) subunit, which leads to familial juvenile epilepsy [12,13]. The heterozygous α1(A322D) knockin mice developed absence seizures with reduced miniature inhibitory postsynaptic currents [36]. Molecular experiments showed that the A322D mutation in TM3 (**Fig 1B**) leads to an unstable transmembrane segment and causes the misfolding and fast elimination of the mutant subunit [13]. Hydrophobic residues at sites homologous to the A322 position in α1 also reside in the β2, β3 and γ2 subunits (**Fig 1C**), consistent with the report that the free energy cost of inserting the TM3 into the membrane has a linear correlation with the hydrophobicity at the A322 position [13]. Here, we aimed to find out a way to restore its functional surface expression. Previously, we showed that application of suberoylanilide hydroxamic acid (SAHA), a FDA-approved drug that crosses the blood-brain barrier, partially restores the function of α1(A322D)β2γ2 GABA_A_ receptors [37]. Furthermore, the Keramidas group demonstrated that SAHA treatment restored the mutant receptor kinetics similar to wild type (WT) [38]. SAHA treatment enhanced the folding and trafficking of the α(A322D) subunit post-translationally by promoting BiP and calnexin-assisted folding in the ER in addition to its role in increasing the transcription of the α1(A322D) subunit [37]. These results indicate that directly operating the ER folding environment is a promising way to restore the forward trafficking of the α1(A322D) subunit.

**Fig 2:**
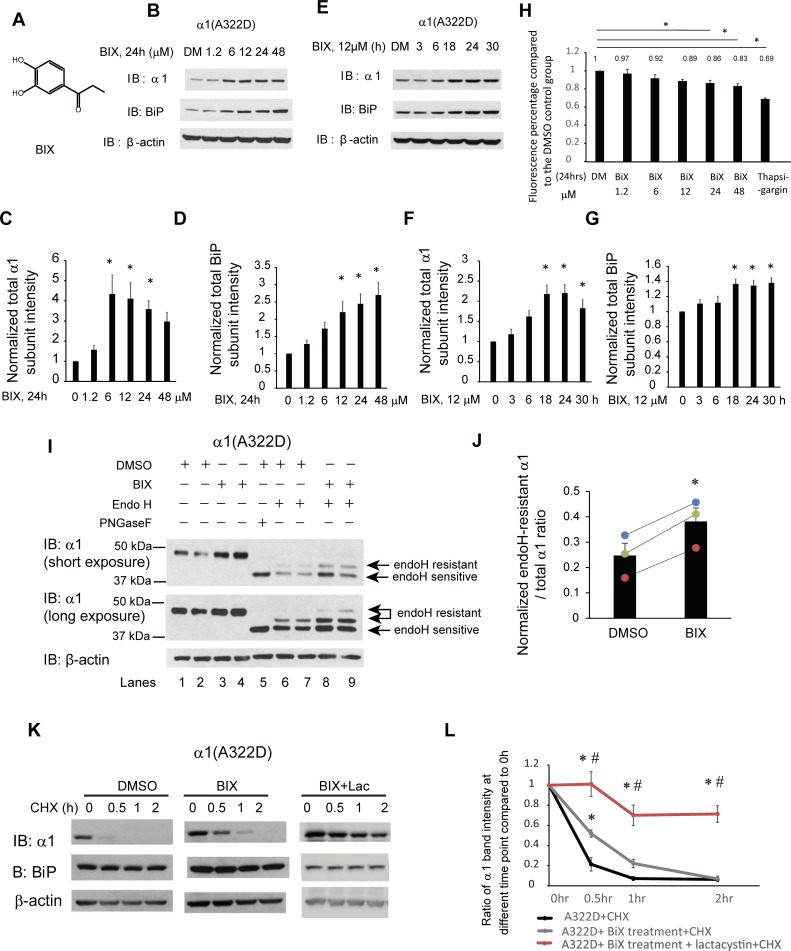
BIX, a potent BiP inducer, enhances the folding and trafficking and reduces the degradation of α1(A322D) subunits. (**A**) Chemical structure of BIX. (**B-D**) Dose response of BIX treatment in regulating α1(A322D) total protein level. HEK293T cells stably expressing α1(A322D)β2γ2 GABA_A_ receptors were treated with BIX at the indicated concentrations or the vehicle control DMSO in the cell culture media for 24 h. Cells were then lysed and subjected to SDS-PAGE and Western blot analysis (**B**). Normalized band intensities for α1(A322D) subunits and BiP are shown in (**C**) and (**D**) (n = 8). (**E-G**) Time course of BIX treatment in regulating α1(A322D) total protein level. HEK293T cells stably expressing α1(A322D)β2γ2 GABA_A_ receptors were treated with BIX (12 μM) for the indicated time. Cells were then lysed and subjected to SDS-PAGE and Western blot analysis (**E**). Normalized band intensities for α1(A322D) subunits and BiP are shown in (**F**) and (**G**) (n = 5). (**H**) HEK293T cells stably expressing α1(A322D)β2γ2 GABA_A_ receptors were plated into a 96-well plate on day 1. Cells were then treated with BIX at the indicated concentrations or the vehicle control DMSO in the cell culture media for 24 h. One groups of HEK293T cells stably expressing α1(A322D)β2γ2 GABA_A_ receptors are treated with thapsigargin (2 μM, 7h) as cell toxicity positive control. Resazurin (0.15mg/ml dissolved in DPBS) is added to cells 1.5 h before plate reading. Fluorescence signal at 560 nm excitation / 590 nm emission is measured. The ratios of fluorescence signal in the DMSO treatment group to treatment groups is shown in (**H**) (n = 4, one-way ANOVA). (**I**) HEK293T cells expressing α1(A322D)β2γ2 receptors were treated with BIX (12 μM, 24 h) or DMSO vehicle control. Then cells were lysed, and total proteins were extracted. Total cellular proteins were incubated with or without endoglycosidase H enzyme (endo H) or peptide-N-glycosidase F (PNGase F) for 1h at 37°C and then subjected to SDS-PAGE and Western blot analysis. Endo H resistant α1 subunit bands (top arrows, lanes 6–9) represent properly folded, post-ER α1 subunit glycoforms that traffic at least to the Golgi compartment, whereas endo H sensitive α1 subunit bands (bottom arrow, lanes 6–9) represent immature α1 subunit glycoforms that are retained in the ER. The PNGase F enzyme cleaves between the innermost N-acetyl-D-glucosamine and asparagine residues from N-linked glycoproteins, serving as a control for unglycosylated α1 subunits (lane 5). The ratio of endo H resistant α1 / total α1, which was calculated from endo H-resistant band intensity / (endo H-resistant + endo H-sensitive band intensity), serves as a measure of trafficking efficiency of the α1(A322D) subunit. Quantification of this ratio after endo H treatment (lanes 6–9) is shown in (**J**) (n = 3, paired t-test). (**K**) HEK293T cells stably expressing α1(A322D)β2γ2 receptors were either treated with DMSO vehicle control, or BIX (12 μM, 24 h) or BIX (12 μM, 24 h) and lactacystin (2.5μM, 24h). Cycloheximide (150 μg/ml), a protein synthesis inhibitor, was added to different cell groups for 0, 0.5 hr, 1 hr, and 2 hrs. Cells were then lysed and subjected to SDS-PAGE and western blot analysis. The quantitation results are shown in (**L**) (n = 5, one-way ANOVA followed by Fisher test, *, p<0.05 between DMSO vehicle control and treatment groups, #, p<0.05 between BIX group and BIX + Lac group). Statistical significance was evaluated using one-way ANOVA followed by post-hoc Tukey test in (**C**), (**D**), (**F**), and (**G**). *, p<0.05. Error bar = SEM.

The ER proteostasis network regulates the ER folding capacity to assure that newly synthesized proteins achieve their native three-dimensional structures in the crowded, oxidative folding environments. The ER proteostasis is mainly monitored by the unfolded protein response (UPR) [39,40]. The UPR senses the ER proteostasis environment using three integrated signaling pathways: inositol-requiring protein-1 (IRE1), activating transcription factor 6 (ATF6), and PKR-like ER kinase (PERK) [39,40]. Because many of the physiologically important proteins fold in the ER, operating the UPR pathway offers great promise to change the fate of pathogenic proteins that are associated with protein misfolding diseases [41,42]. Researchers are utilizing the UPR activation as a therapeutic strategy to ameliorate various protein conformational diseases, including lysosomal storage diseases [43], transthyretin amyloidosis and light chain amyloidosis [44,45], retinitis pigmentosa [46,47], α1 antitrypsin deficiency [48], and familial forms of amyotrophic lateral sclerosis [49]. However, the question of whether the UPR activation restores proteostasis of pathogenic, misfolded ion channel proteins remains unclear. Moreover, BiP (binding immunoglobulin protein), an ER resident HSP70 family protein, is a prominent target of ATF6 and IRE1 activation [50]. A specific BiP activator, BIX (BiP protein inducer X, 1-(3,4-dihydroxy-phenyl)-2-thiocyanate-ethanone) **(Fig 2A**), induces the BiP expression without disturbing other ER chaperone expression [51]. Here, we evaluated how regulating ER proteostasis network by applying BIX or activating two major UPR branches (ATF6 and IRE1) influences the surface trafficking of the α1(A322D) subunit.

## Results

### BIX treatment promotes the ER-to-Golgi trafficking and reduces the degradation of the mutant α1(A322D) subunit

Previously, Connolly et al. demonstrated that BiP interacts with α1 subunits of GABA_A_ receptors [27], and we demonstrated that BiP overexpression is sufficient to enhance the folding and forward trafficking of the α1(A322D) subunits [37]. The small molecule BIX induces BiP expression through an ATF6-dependent mechanism, but does not substantially induce expression of other ATF6 target genes, such as Grp94 and PDIA4 in SK-N-SH neuroblastoma cells [51,52]. Previous studies showed that cerebral pretreatment with BIX in ischemia mice protects neurons from ER stress-induced cell death by enhancing BiP level [51]. Therefore, we evaluated BIX’s effect on BiP and α1(A322D) protein levels in HEK293T cells stably expressing α1(A322D)β2γ2 receptors. Dose-response analysis showed that BIX (24 h treatment) increased the BiP protein levels significantly at concentrations of 12, 24, and 48 μM (**Fig 2B**, quantification of the BiP band intensity shown in **Fig 2D**). Moreover, BIX treatment increased the total α1(A322D) subunit significantly at 6 μM and this effect plateaued from 6 μM to 24 μM (**Fig 2B**, quantification of the α1 band intensity shown in **Fig 2C**). Time-course study demonstrated that BIX’s effect on increasing BiP and total α1(A322D) subunit level was achieved as early as 18 h and plateaued from 18 h to 30 h (**Fig 2E**, quantification of the α1 band intensity shown in **Fig 2F** and the BiP band intensity shown in **Fig 2G**). This time scale is possibly due to the involvement of protein maturation, such as protein folding and trafficking. Resazurin cell toxicity assay demonstrated that single-dose applications of BIX for 24 h did not induce significant toxicity to cells at concentrations no more than 12 μM (**Fig 2H**). Higher concentrations (24 μM and 48 μM) of BIX led to reduced cell viability (**Fig 2H**), which could account for the decreased total α1 protein levels at such concentrations (**Fig 2C**). Therefore, we used the optimal incubation condition of BIX (12 μM, 24 h) for the following experiments. Furthermore, because the total expression level of GABA_A_ receptors could adjust the capacity of the ER proteostasis network prior to the treatment, we evaluated whether BIX administration was effective in the context of different protein synthesis load. HEK293T cells were transiently transfected with 0.15 μg of α1(A322D), β2, and γ2 plasmids or 0.25 μg of them and treated with BIX. Western blot analysis demonstrated that BIX treatment increased the total α1(A322D) protein levels in both cases (**S1 Fig**). It appeared that BIX treatment led to less increase of the total α1(A322D) protein in the 0.25 μg transfection group compared to that in the 0.15 μg transfection group (**S1 Fig**), indicating that BIX’s efficacy partially depended on the protein synthesis load. Therefore, care must be taken not to overload the proteostasis network when testing the effect of added proteostasis regulators.

Next we determined whether BIX promoted the folding and the ER-to-Golgi trafficking of the α1(A322D)β2γ2 receptors. We first performed the endoglycosidase H (endo H) enzyme digestion experiment, which tracks the forward trafficking of a glycoprotein from the ER to the Golgi. Endo H cleaves the mannose-rich core glycans (the ER glycoform), which are attached to Asn residues in an Asn-X-Ser/Thr sequon (X can be any residue except proline). But endo H can not eliminate the oligosaccharide chain after glycan remodeling in the Golgi. Therefore, this assay indirectly evaluates whether a glycoprotein is properly folded in the ER. The α1 subunit has two *N*-linked glycosylation sites at Asn38 and Asn138. The peptide-N-glycosidase F (PNGase F) enzyme cleaves between the innermost N-acetyl-D-glucosamine and Asn residues from *N*-linked glycoproteins, serving as a control for unglycosylated α1 subunits (**Fig 2I**, lane 5). Endo H digestion experiments demonstrated that BIX treatment (12 μM, 24 h) clearly increased the endo H-resistant α1(A322D) band intensity (**Fig 2I**, cf. lanes 8 and 9 to lanes 6 and 7) as well as the ratio of endo H resistant / total α1(A322D) significantly (**Fig 2I**, quantification shown in **Fig 2J**), indicating that BIX treatment enhances the trafficking efficiency of the α1(A322D) subunit from the ER to Golgi, and consequently, more properly folded α1(A322D) proteins are able to reach at least to the Golgi.

Previous study showed that A322D mutation dramatically increases its degradation rate with a half-life as short as of 23 min compared to WT α1 subunits with a half-life of more than 90 min [13,37]. We then performed cycloheximide-chase experiments to evaluate the degradation of α1(A322D) subunits by applying cycloheximide to inhibit the protein synthesis in HEK293T cells stably expressing α1(A322D)β2γ2 receptors. BIX treatment significantly increased the remaining α1(A322D) subunits from 26.7% to 51.3% after 0.5 h cycloheximide chase, and from 9% to 23% after 1 h cycloheximide chase (p<0.05) (**Fig 2K**, quantification shown in **Fig 2L**). As a result, BIX treatment reduced the degradation of α1(A322D) subunits, consistent with the increased total α1(A322D) protein levels. Moreover, addition of lactacystin, a potent proteasome inhibitor, with BIX treatment further attenuated the degradation of α1(A322D) (**Fig 2K**, quantification shown in **Fig 2L**), indicating that indeed the cycloheximide chase experiments reflected that the ERAD plays the most important role in the degradation of α1(A322D).

### BIX treatment promotes the functional surface expression of α1(A322D) subunit

**Fig 3:**
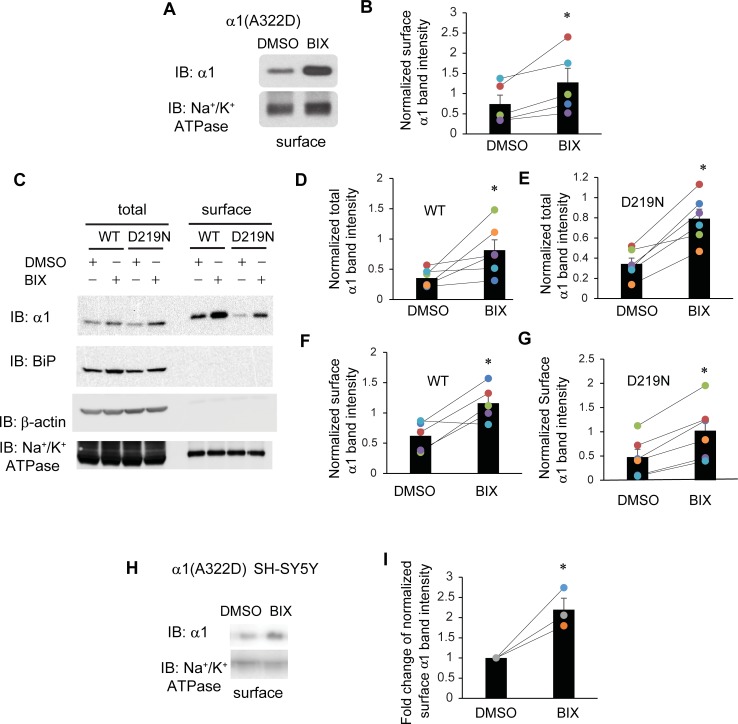
BIX enhances the surface expression of α1 subunit variants of GABA_A_ receptors. (**A**) HEK293T cells expressing α1(A322D)β2γ2 receptors were treated with BIX (12 μM, 24 h) or DMSO vehicle control. Then the cell surface proteins were tagged with biotin using membrane-impermeable biotinylation reagent sulfo-NHS SS-Biotin. Biotinylated surface proteins were affinity-purified using neutravidin-conjugated beads and then subjected to SDS-PAGE and Western blot analysis. The Na^+^/K^+^-ATPase serves as a surface protein loading control. Quantification of normalized surface α1(A322D) protein levels to the Na^+^/K^+^-ATPase controls is shown in (**B**) (n = 5, paired t-test). (**C**) HEK293T cells expressing α1β2γ2 receptors or α1(D219N)β2γ2 receptors were treated as in (**A**). Quantification of normalized total and surface WT α1 protein levels is shown in (**D** & **F**) (n = 6 for total and n = 5 for surface, paired t-test). Quantification of normalized total and surface α1(D219N) protein levels is shown in (**E** & **G**) (n = 6 for total and surface, paired t-test). (**H**) SH-SY5Y cells stably expressing α1(A322D)β2γ2 receptors were treated with BIX (12 μM, 24 h) or DMSO vehicle control. Then surface biotinylation assay was performed as in (**A**). Quantification of normalized surface α1(A322D) protein levels is shown in (**I**) (n = 3, two tailed student t-test). * *p* < 0.05.

We next asked whether the upregulated α1(A322D) proteins afforded by BIX administration reach the plasma membrane for their function. Surface biotinylation experiments demonstrated that BIX treatment increased α1(A322D) subunit in the plasma membrane significantly in HEK293T cells (**Fig 3A** and **3B**). We further tested the BIX effect on WT α1 subunit and another misfolding-prone mutant α1(D219N) subunit [53,54]. Surface biotinylation experiments showed that BIX treatment increased both the total and plasma membrane protein levels for WT α1 and α1(D219N) subunits significantly (**Fig 3C,** quantification shown in **Fig 3D–3G**), indicating that BIX treatment can enhance the surface trafficking of a variety of misfolding-prone subunits. The positive effect of BIX on WT α1 subunits was because these WT membrane proteins do not fold and assemble efficiently in the ER and part of them are degraded by the ERAD pathway [55]. We also tested the effect of BIX treatment on the surface trafficking of α1(A322D) subunits in human SH-SY5Y neuroblastoma cells stably expressing α1(A322D)β2γ2 receptors. Surface biotinylation experiments showed that BIX treatment significantly enhanced the surface expression of α1(A322D) subunits 2.2-fold (**Fig 3H**, quantification shown in **Fig 3I**).

**Fig 4:**
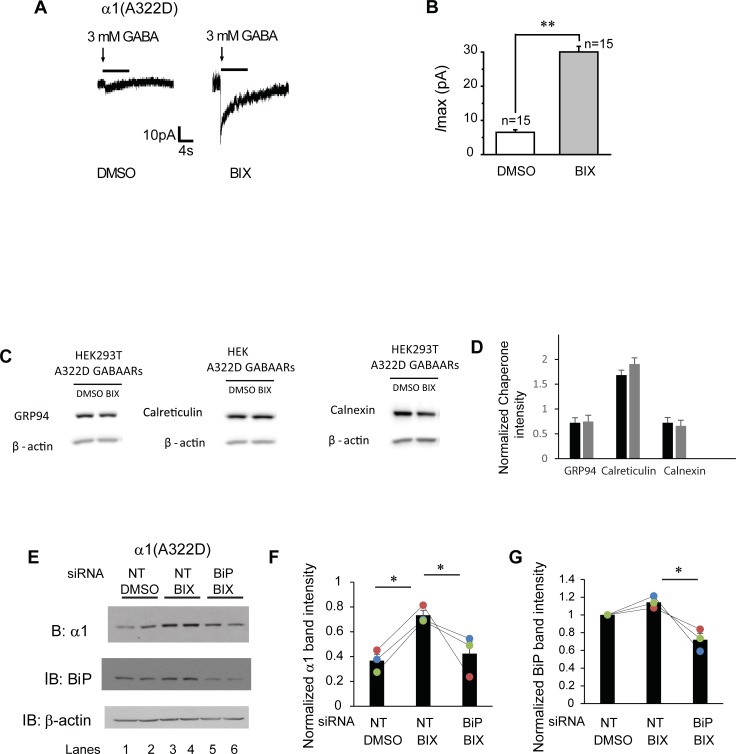
BIX enhances the function of α1(A322D)β2γ2 receptors. (**A**) Representative whole-cell patch clamping recording traces in monoclonal HEK293T cells stably expressing α1(A322D)β2γ2 GABA_A_ receptors. Cells were treated with BIX (12 μM, 24h) or DMSO before voltage clamping. GABA (3mM) was applied to induce chloride currents with a holding potential of -60 mV. Quantification of the peak currents (*I*max) is shown in (**B**). The number of patched cells in each group is shown on the top of the bar. pA: picoampere. (**C** and **D**) HEK293T cells expressing α1(A322D)β2γ2 receptors were treated with BIX (12μM, 24h). The cell lysates were then subjected to SDS-PAGE and Western blot analysis using corresponding antibodies (**C**). Quantification of normalized total cellular chaperone protein expression levels is shown in (**D**) (n = 4, paired t-test). (**E**) HEK293T cells stably expressing α1(A322D)β2γ2 receptors were treated with non-targeting (NT) or BiP siRNA for 48 hrs. Cells were then treated either with BIX (12 μM) or DMSO vehicle control for another 24 hrs. Cells were then lysed and subjected to SDS-PAGE and western blot analysis. The quantitation results of α1(A322D) and BiP are shown in (**F**&**G**) (n = 3, one-way ANOVA). * *p* < 0.05; ** *p* < 0.01.

We then tested whether the increased surface expression of α1(A322D) subunits is functional using whole-cell voltage-clamping electrophysiology to record GABA-induced chloride currents. To minimize the variation in the recording of GABA-induced currents among different cells, we generated monoclonal HEK293T cells stably expressing α1(A322D)β2γ2 GABA_A_ receptors. To achieve that, we subcloned the α1(A322D) into a pIRES2-EGFP bicistronic vector, which would allow the simultaneous expression of α1 subunits and EGFP separately but from the same RNA transcript. This enabled us to select GFP-positive single cells for electrophysiology recording. The peak chloride current in response to GABA (3 mM) was only 6.0 pA in untreated HEK293T cells expressing α1(A322D)β2γ2 GABA_A_ receptors (**Fig 4A**), indicating that essentially no functional channels reside in the plasma membrane. Strikingly, BIX treatment significantly increased this current to 30 pA (**Fig 4A**, quantification shown in **Fig 4B**), indicating that BIX partially corrected the function of this pathogenic mutant GABA_A_ receptors on the plasma membrane. Previously, we showed that GABA-induced peak chloride current in HEK293T cells expressing WT GABA_A_ receptors was 138 pA [37]. Therefore, the peak current for BIX-rescued α1(A322D)β2γ2 receptors amounted to 22% of that for WT receptors, greater than that for SAHA-rescued mutant receptors [37]. A recent report revealed that despite the relatively modest peak current increase, SAHA treatment restored the receptor kinetics in heterosynaptic cultures harboring the α1(A322D) mutation that were indistinguishable from those harboring the WT receptors [38]. Therefore, although the physiological relevance of the BIX treatment remains to be established, since previous studies showed that BIX protects neurons from stress-induced cell death [51], BIX is promising to be further developed to correct GABA_A_ receptor misfolding diseases.

Next we determined the influence of BIX on the ER proteostasis network. BIX treatment (12 μM, 24 h) did not significantly change the expression levels of several major ER chaperones, including Grp94, calreticulin and calnexin in HEK293T cells stably expressing α1(A322D)β2γ2 receptors (**Fig 4C**, quantification shown in **Fig 4D**), indicating that BIX’s prominent role is to induce BiP expression. Therefore, we evaluated whether the BIX’s effect on α1(A322D) subunits depends on the BiP expression level. As expected, BIX treatment increased the α1(A322D) protein levels in the non-targeting siRNA treatment groups in HEK293T cells stably expressing α1(A322D)β2γ2 receptors (**Fig 4E**, cf. lanes 3 and 4 to lanes 1 and 2) (α1(A322D) subunits quantification shown in **Fig 4F** and BiP quantification shown in **Fig 4G**). After BiP expression was depleted with BiP siRNA treatment, BIX treatment did not significantly change the α1(A322D) protein levels (**Fig 4E**, cf. lanes 5 and 6 to lanes 1 and 2, quantification shown in **Fig 4F**). The data indicated that the effect of BIX on α1(A322D) subunits is by modulating BiP expression level.

### Activation of the ATF6 pathway promotes the forward trafficking of the mutant α1(A322D) subunit

**Fig 5:**
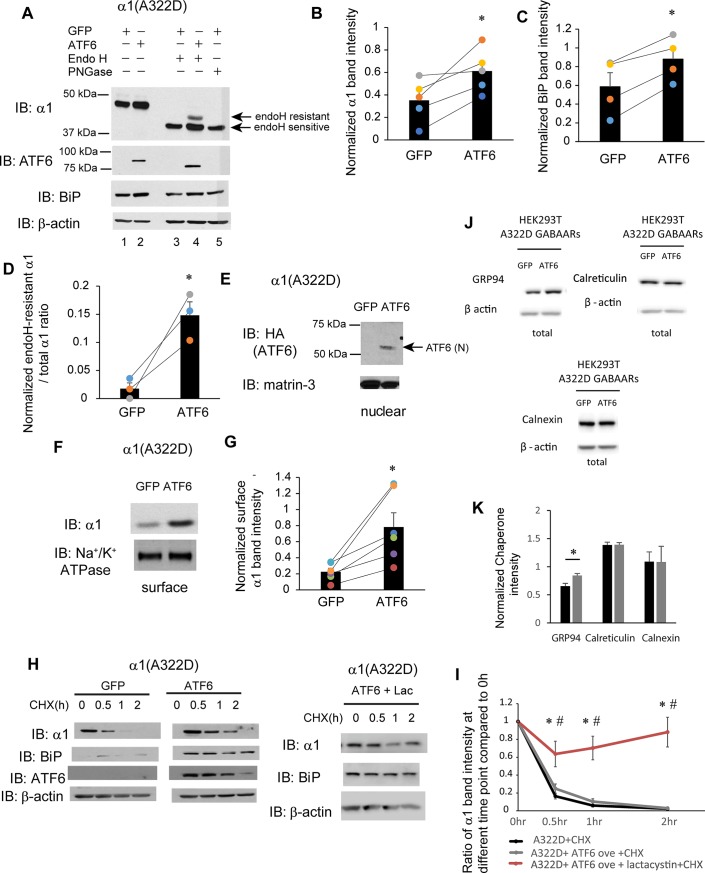
ATF6 activation promotes the forward trafficking of α1(A322D) subunit of GABA_A_ receptors. (A) HEK293T cells expressing α1(A322D)β2γ2 receptors were transiently transfected with GFP or HA-tagged full-length ATF6α plasmids. Forty-eight hrs post transfection, cells were lysed, and total proteins were extracted. Total cellular proteins were incubated with or without endoglycosidase H enzyme (endo H) or peptide-N-glycosidase F (PNGase F) for 1h at 37°C and then subjected to SDS-PAGE and Western blot analysis using corresponding antibodies. Endo H resistant v1 subunit bands (top arrow, lane 4) represent properly folded, post-ER α1 subunit glycoforms that traffic at least to the Golgi compartment, whereas endo H sensitive α1 subunit bands (bottom arrow, lanes 3 and 4) represent immature α1 subunit glycoforms that are retained in the ER. The PNGase F enzyme cleaves between the innermost N-acetyl-D-glucosamine and asparagine residues from N-linked glycoproteins, serving as a control for unglycosylated α1 subunits (lane 5). Quantification of total cellular protein expression levels of α1 and BiP is shown in (**B**) and (**C**) (n = 5 for α1 and n = 4 for BiP, paired t-test). Quantification of the ratio of endo H resistant α1 / total α1 is shown in (**D**) (n = 3, paired t-test). (**E**) Cells were treated as in (**A**). Forty-eight hrs post transfection, the nuclear fractions were extracted and subject to SDS-PAGE. ATF6 (N) is the cleaved, activated N-terminal ATF6 in the nucleus. Matrin-3 serves as a nuclear protein loading control. (**F**) HEK293T cells were treated as in (**A**). Forty-eight hrs post transfection, the cell surface proteins were tagged with biotin using membrane-impermeable biotinylation reagent sulfo-NHS SS-Biotin. Biotinylated surface proteins were affinity-purified using neutravidin-conjugated beads and then subjected to SDS-PAGE and Western blot analysis. The Na^+^/K^+^-ATPase serves as a surface protein loading control. Quantification of normalized surface α1(A322D) protein levels is shown in (**G**) (n = 6, paired t-test). (**H**) HEK293T cells expressing α1(A322D)β2γ2 receptors were either transfected with GFP control, or ATF6, or transfected with ATF6 and treated with lactacystin (2.5μM for 24h). Cycloheximide (150 μg/ml), a protein synthesis inhibitor, was added to different cell groups for 0, 0.5 hr, 1 hr, and 2 hrs. Cells were then lysed and subjected to SDS-PAGE and western blot analysis. The quantitation results are shown in (**I**) (n = 5, one-way ANOVA followed by Fisher test, *, p<0.05 between GFP control and ATF6 + Lac group, #, p<0.05 between ATF6 group and ATF6 + Lac group). (**J** and **K**) HEK293T cells expressing α1(A322D)β2γ2 receptors were either transfected with GFP or ATF6 for 48h. The cell lysates were then subjected to SDS-PAGE and Western blot analysis using corresponding antibodies (**J**). Quantification of total cellular chaperone protein expression levels is shown in (**K**) (n = 4, paired t-test). *, p<0.05.

Because BiP is one major ER chaperone whose expressions are regulated by UPR [50], we next determined the effect of activating the UPR on the maturation of the α1(A322D) subunits. Among the three UPR arms, activation of ATF6 pathway and IRE1 pathway is known to enhance the ER folding capacity, whereas the PERK pathway activation often leads to apoptosis and reduces the protein synthesis when the pathway is continuously turned on [40]. Therefore, we focused on the ATF6 arm and IRE1 arm. We first examined how ATF6 influenced the maturation of α1(A322D) subunits. ATF6 has two homologs: ATF6α and ATF6β. The role of ATF6α in the UPR has been well-defined, whereas ATF6β has not been thoroughly studied. Here, we overexpressed full-length HA-tagged ATF6α in HEK293T cells stably expressing α1(A322D)β2γ2 GABA_A_ receptors. Western blot analysis showed that, as expected [56], ATF6α overexpression increased total protein expression level of both full-length ATF6α and BiP, an ATF6 target gene (**Fig 5A**, cf. lane 2 to 1, quantification of the BiP band intensity shown in **Fig 5C**). Furthermore, ATF6α overexpression generated the cleaved, activated N-terminal form of ATF6 (N) in the nucleus (**Fig 5E**), confirming the activation of the ATF6 pathway in HEK293T cells. As a result, this operation led to a substantial 1.74-fold increase of the total protein level of α1(A322D) subunits (**Fig 5A**, quantification shown in **Fig 5B**).

In order to evaluate whether ATF6 activation promotes the folding and ER-to-Golgi trafficking of the mutant α1(A322D) subunits, we performed the endoglycosidase H (endo H) enzyme digestion experiment. Endo H digestion produced a single α1(A322D) band at the molecule weight size of the unglycosylated subunit (**Fig 5A**, lane 3), which is designated as endo H-sensitive, indicating that the majority of the α1(A322D) subunit is retained in the ER for fast degradation. ATF6 overexpression led to a clear visualization of a strong post-ER α1(A322D) subunit band (**Fig 5A**, lane 4, top endo H-resistant band). Moreover, the ratio of the mature α1(A322D) subunit (endo H-sensitive band) to total α1(A322D) subunit (the sum of endo H-sensitive and endo H-resistant band) is increased (quantification shown in **Fig 5D)**. This result indicates that activation of the ATF6 pathway enhanced the folding and ER-to-Golgi trafficking of the α1(A322D) subunits. Furthermore, surface biotinylation experiments showed that ATF6 overexpression substantially increased the surface protein level of the α1(A322D) subunits (**Fig 5F**, quantification shown in **Fig 5G**).

Interestingly, the cycloheximide chase experiment revealed that ATF6 activation did not significantly alter the degradation rate of α1(A322D) subunits (**Fig 5H**, quantification shown in **Fig 5I**). As a control to evaluate whether ATF6 activation induced autophagy as a major way to degrade α1(A322D), we incubated lactacystin, a potent proteasome inhibitor, in HEK293T cells overexpressing ATF6. This operation further slowed down the degradation of α1(A322D) (**Fig 5H**, quantification shown in **Fig 5I**), indicating that the cycloheximide chase experiments were mostly a result of ERAD. Possibly, because ATF6 activation has profound effect on ER proteostasis network, the ERAD pathway could be elevated in addition to the folding enhancement. Therefore, we evaluated how ATF6 activation regulated major ER chaperones in HEK293T cells expressing α1(A322D)β2γ2 receptors. Genetic activation of ATF6 significantly increased the total protein level of Grp94, but not calreticulin or calnexin (**Fig 5J**, quantification in **Fig 5K**). Previously, we demonstrated that Grp94 targets α1(A322D) to the ERAD pathway [55]. As such, elevated Grp94 expression could lead to enhanced ERAD and contribute to the apparently unchanged stability of α1(A322D) after ATF6 activation.

In summary, the above experiments clearly demonstrated that ATF6 activation promotes the folding of the α1(A322D) subunits and their forward trafficking from the ER to Golgi and onward to the plasma membrane.

### Activating the IRE1 pathway increases the surface level of the mutant α1(A322D) subunit

**Fig 6:**
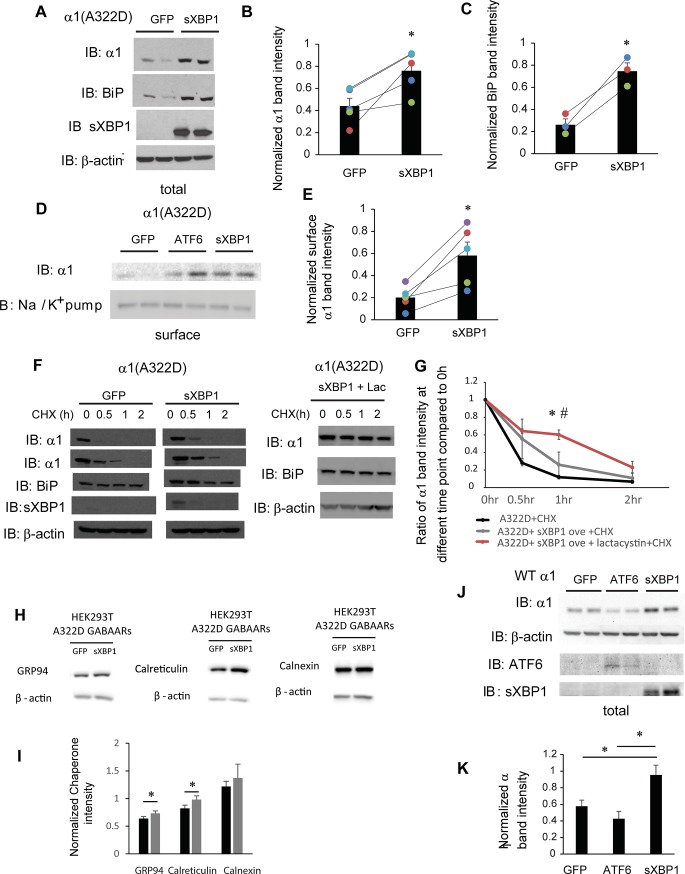
IRE1 activation increases the surface expression of α1(A322D) subunit of GABA_A_ receptors. (**A**) HEK293T cells expressing α1(A322D)β2γ2 receptors were transiently transfected with GFP or XBP1-s (spliced XBP1) plasmids. Forty-eight hrs post transfection, cells were lysed, and total proteins were extracted. The cell lysates are then subjected to SDS-PAGE and Western blot analysis using corresponding antibodies. Quantification of total cellular protein expression levels of α1 and BiP is shown in (**B** & **C**) (n = 5 for α1 and n = 3 for BiP, paired t-test). (**D**) HEK293T cells were treated as in (**A**). Forty-eight hrs post transfection, the cell surface proteins were tagged with biotin using membrane-impermeable biotinylation reagent sulfo-NHS SS-Biotin. Biotinylated surface proteins were affinity-purified using neutravidin-conjugated beads and then subjected to SDS-PAGE and Western blot analysis. The Na^+^/K^+^-ATPase serves as a surface protein loading control. Quantification of normalized surface protein expression levels of α1 is shown in (**E**) (n = 5, paired t-test). (**F**) HEK293T cells expressing α1(A322D)β2γ2 receptors were either transfected with GFP control, or XBP-s or transfected with XBP-s and treated with lactacystin (2.5 μM for 24h). Cycloheximide (150 μg/ml), a protein synthesis inhibitor, was added to different cell groups for 0, 0.5 hr, 1 hr, and 2 hrs. Cells were then lysed and subjected to SDS-PAGE and western blot analysis. The quantitation results are shown in (**G**) (n = 3, one-way ANOVA followed by Fisher test, *, p<0.05 between control group and XBP1-s + Lac group, #, p<0.05 between XBP1-s group and XBP1-s + Lac group). (**H** and **I**) HEK293T cells expressing α1(A322D)β2γ2 receptors were either transfected with GFP or XBP1s for 48h. The cell lysates were then subjected to SDS-PAGE and Western blot analysis using corresponding antibodies (**H**). Quantification of total cellular chaperone protein expression levels is shown in (**I**) (n = 4, paired t-test). *, p<0.05. (**J**) HEK293T cells expressing WT α1β2γ2 receptors were transfected with GFP, ATF6 or XBP1s plasmids. Forty-eight hrs post transfection, cells were lysed, and total proteins were extracted and subject to SDS-PAGE and Western blot analysis. Quantification of total cellular protein expression levels of α1 is shown in (**K**) (n = 3, paired t-test using adjusted p values). *, p<0.05.

IRE1, an ER transmembrane kinase/endoribonuclease, responds to misfolded proteins in the ER by oligomerization and autophosphorylation [40]. IRE1 activation cleaves the inactivated form of XBP1 (X-box binding protein 1) mRNA to produce spliced XBP1 mRNA, which encodes the transcriptional factor XBP1s. Here, we evaluated how IRE1 pathway activation affects the maturation of α1(A322D) subunits. In order to activate the IRE1 pathway, we overexpressed active form of transcriptional factor XBP1s in HEK293T cells expressing α1(A322D)β2γ2 GABA_A_ receptors. Western blot analysis showed that XBP1s overexpression increased total protein expression level of both XBP1s and BiP (**Fig 6A**, quantification of the BiP band intensity shown in **Fig 6C**). XBP1s overexpression led to a substantial 1.72-fold increase of the total protein level of α1(A322D) subunits (**Fig 6A**, quantification shown in **Fig 6B**).

Furthermore, surface biotinylation experiments showed that XBP1s overexpression substantially increased the surface protein level of the α1(A322D) subunits (**Fig 6D,** quantification shown in **Fig 6E**), indicating that IRE1 activation promotes the surface expression of the α1(A322D) subunit. The cycloheximide chase experiment showed that although IRE1 pathway activation group has 48.5% α1(A322D) left compared to 26.5% of control group after 0.5 hr chase, and has 22.3% α1(A322D) left compared to 15.4% of control group after 1 hr chase, there is no significant difference in the remaining α1(A322D) at all the time points between GFP control group and XBP1s overexpression group (**Fig 6F**, quantification shown in **Fig 6G**, n = 4). To determine whether XBP1s activation induced autophagy as an alternative to degrade α1(A322D), we treated HEK293T cells overexpressing XBP1s with lactacystin, a potent proteasome inhibitor. Clearly, lactacystin incubation further reduced the degradation of α1(A322D) (**Fig 6F**, quantification shown in **Fig 6G**), indicating that the cycloheximide chase experiments mostly resulted from ERAD. To explore the possible reasons that lead to enhanced surface trafficking without increased stability, we evaluated how IRE1 activation influenced major ER chaperones in HEK293T cells expressing α1(A322D)β2γ2 receptors. XBP1s overexpression significantly increased the total protein level of Grp94 and calreticulin, but not calnexin (**Fig 6H**, quantification in **Fig 6I**). Accordingly, the unaltered stability of α1(A322D) after IRE1 activation could result from the increased Grp94 protein level, which promoted its ERAD targeting [55].

In addition, XBP1s overexpression significantly increased the total protein level of WT α1 subunits in HEK293T cells expressing WT receptors, whereas ATF6 overexpression did not (**Fig 6J**, quantification shown in **Fig 6K**). The apparently unchanged WT α1 protein levels after ATF6 activation could come from the competition between promoted folding, such as from BiP upregulation, and enhanced ERAD, such as from Grp94 upregulation. The different effect between ATF6 and IRE1 activation could be because ATF6 and IRE1 have overlapping, but different downstream targets [50]. This result also suggests that ATF6 activation could have a more selective effect on the misfolding-prone mutant receptors over WT receptors, and thus merits further development.

## Discussion

Here, we used well-characterized misfolding-prone α1(A322D) subunits to evaluate how remodeling the ER proteostasis network influences their maturation by applying a chemical that specifically enhances the expression level of ER-resident HSP70 family protein BiP or enhancing the folding capacity through activating two major UPR branches: the ATF6 pathway or the IRE1 pathway. Our results showed that the specific BiP activator, BIX, promotes the folding, trafficking and importantly functional surface expression of α1(A322D) (**Figs 2I**, **3A**, and **4A**). BIX also enhances surface expression level of α1(D219N) (**Fig 3C**). Because GABA_A_ receptor subunits need to assemble properly into defined heteropentamers on the ER membrane before they exit the ER [27,57,58], the improved surface trafficking of α1(A322D) and α1(D219N) by BIX treatment could arise from enhanced subunit interactions and subunit assembly efficiency on the ER membrane. Currently, many of the fundamental questions about how the proteostasis network regulates the assembly process of multi-subunit membrane proteins in the ER remain to be answered. Therefore, the characterization of the chaperone-assisted assembly process of these membrane proteins could open a new way to promote their surface trafficking. BIX has significant neuron-protective effects in brain ischemia mice model [51,52], which makes BIX a promising candidate to be further developed as a strategy to ameliorate idiopathic epilepsy resulting from GABA_A_ receptor misfolding. Furthermore, previously we showed that inhibition of valosin-containing protein (VCP) and SAHA treatment are able to enhance functional surface expression of α1(A322D) mutant receptors by attenuating the ERAD, increasing the synthesis, and/or promoting folding and trafficking of mutant receptors [37,59]. Therefore, a combination usage of BIX and these mechanistically distinct proteostasis regulators could potentially improve the functional rescue of quite malignant mutant receptors, which merits future investigations. In addition, pharmacological chaperones directly bind to their target protein to stabilize it in a protein-pharmacological chaperone state [60]. It was reported that pharmacological chaperones up-regulated the surface expression of several Cys-loop receptors, including neuronal acetylcholine receptors [61] and GABA_A_ receptors [62]. Therefore, co-application of pharmacological chaperones and BIX has the promise to further promote functional surface expression of mutant receptors.

We further demonstrated that activating either ATF6 pathway or IRE1 pathway increases the surface expression of pathogenic GABA_A_ receptors carrying the A322D mutation in the α1 subunit. Both the IRE1 pathway and the ATF6 activation remodel the ER proteostasis network to enhance the folding of the α1(A322D) subunit, enabling its successful transport to the plasma membrane. Interestingly, previous studies showed that activating ATF6 reduces the secretion and extracellular aggregation of the amyloidogenic protein transthyretin by enhancing ER quality control stringency and thus ERAD [44]. Here, we showed that ATF6 activation enhances the forward trafficking of mutant α1(A322D) subunits. However, ATF6 activation does not influence the degradation rate of total mutant α1(A322D) subunits. This may indicate that ATF6 activation favor the folding and forward trafficking of mutant α1(A322D) subunits. Previous study also showed that the activation of IRE1 pathway specifically without affecting two other UPR pathways by using a chemical control system promotes the degradation of misfolding mutant R21H rhodopsin protein in retinal cells [47]. However, our result showed that XBP1s overexpression does not decrease the degradation rate of α1(A322D) subunits, but promotes their surface expression. It seems that ATF6 pathway or IRE1 pathway activation produces two competing downstream effects: folding enhancement and ERAD enhancement. The net effect on protein trafficking might depend on the specific proteins of interest with a goal to be cytoprotective. Operating ATF6 pathway or IRE1 pathway appears to be a promising strategy to ameliorate protein conformational diseases. ATF6 and IRE1 have selective targets among the ER proteostasis network. A comprehensive analysis of chaperones and folding enzymes that contribute to ATF6’s and IRE1’s effect needs to be carried out in the future by analyzing the α1(A322D) interactome before and after the activation of ATF6 pathway or IRE1 pathway.

By definition, proteins with misfolded lesions in the ER *l*umen undergo the ERAD-L pathway, whereas proteins with misfolded lesions in the *m*embrane region undergo the ERAD-M pathway [63]. A known ERAD-L substrate is the α1(D219N) subunit because D219N is located in the N terminus ER lumen of the α1 subunit, causing the aggravated degradation in the ER and decreased functional surface expression, whereas a known ERAD-M substrate is the α1(A322D) subunit. BIX treatment enhances the surface trafficking of both ERAD-L substrates (**Fig 3C**) and ERAD-M substrates (**Fig 3A**), which depends on the BiP expression levels. The D219N mutation resides in the ER lumen, and it is understandable that such ERAD-L substrates can be stabilized by ER resident chaperones, such as BiP and calnexin [53]. Consistently, we showed that BIX treatment is sufficient to enhance the surface trafficking of α1(D219N). Why could the surface trafficking of ERAD-M substrates, such as α1(A322D), be rescued by operating the ER proteostasis network, such as BIX treatment and activating ATF6/IRE1? The A322D mutation resides in the middle of TM3 (**Fig 1B**). Because BIX treatment or BiP overexpression is sufficient to enhance the forward trafficking of α1(A322D) subunits [37], one possibility is that BiP is able to stabilize the ER lumen domain of α1(A322D) subunits, allowing more time for the insertion of TM3 into the lipid bilayers. Such a possibility is also supported by our recent report that inhibiting VCP to slow down the ERAD of α1(A322D) subunits can enhance their forward trafficking [59]. Alternatively, BiP is able to directly stabilize the TM3 segment after TM3 is exposed to the ER lumen. The retention of α1(A322D) subunits from aggregation and/or ERAD will permit the re-insertion of TM3 into lipid bilayers.

As for the effect of ATF6 and IRE1 activation, another possibility is that their downstream targets could regulate the insertion of TM3 into the lipid bilayers. There are only a limited number of reports about the physical and/or functional interactions between TM segments and the ER luminal chaperones. One example is the yeast chaperone Lhs1 or its mammalian homolog Grp170 (aka HYOU1), which is a nucleotide exchange factor (NEF) for the ER luminal Hsp70 [64]. Grp170 is a downstream target of both ATF6 and IRE1 [50,65]. Lhs1 selectively targets the α subunit of the epithelial sodium channel (ENaC) for ERAD, which is independent of Lhs1’s NEF function [66]. ENaC needs to assemble into a heterotrimer from α, β, and γ subunits on the ER membrane before its efficient trafficking to the plasma membrane. Co-expression of β and γ subunits with α subunits prevents Lhs1-dependent ERAD of α subunits [67]. Furthermore, the intersubunit interactions through TM domains of the α subunits play a critical role in blocking Lhs1-dependent ERAD. These results indicate that Lhs1 directly or indirectly interacts with the exposed TM domains of the α subunits for ERAD; alternatively, Lhs1 recognizes the ER lumen domains of monomeric α subunits, the conformation of which will be changed upon the subunit assembly process. Another example is the ER luminal UDP-glucose:glycoprotein glucosyltransferase UGGT1, which reglucosylate the partially folded glycoproteins to re-enter the calnexin/calreticulin folding cycles [68]. To study the ERAD-M pathway, a model ERAD-M substrate α1AT_C_ was built by fusing α1-antitrypsin to the C-terminal domain of CD3δ with an Asp residue in the TM segment [69]. It appears that UGGT1 recognizes the conformation that is associated with the TM defect of α1AT_C_, whereas deleting the TM defect of α1AT_C_ blocks its interaction with UGGT1 [69]. It will be of great interest to characterize whether and how such chaperones influence the assembly and degradation of the α1(A322D) subunits and other ERAD-M substrates.

Increasing number of missense mutations in GABA_A_ receptors has been identified to cause epilepsy. Many of them are believed to compromise the protein folding, assembly, and trafficking of the mutant receptors [17]. Our current study provides a proof-of-principle case, showing that correcting protein misfolding is a novel therapeutic strategy for such epilepsies. Further efforts are desired to find out the effectiveness of proteostasis regulators in a variety of pathogenic GABA_A_ receptors with folding or assembly deficiency.

## Materials and methods

### Chemicals, plasmids, and antibodies

BIX (BiP protein inducer X) was obtained from Sigma (#SML1073). Thapsigargin was obtained from Enzo life science (BML-PE180-0001). Lactacystin was obtained from AdipoGen life science (#AG-CN2-0104). Resazurin was obtained from MP biomedicals (#0219548101). The pCMV6 plasmids containing human GABA_A_ receptor α1 (Uniprot no. P14867-1), β2 (isoform 2, Uniprot no. P47870-1), and γ2 (isoform 2, Uniprot no. P18507-2) subunits and the pCMV6 Entry Vector plasmid (pCMV6-EV) were purchased from Origene. A FLAG tag was inserted between Leu31 and Gln32 in the α1 subunit using QuikChange II site-directed mutagenesis Kit (Agilent Genomics); this operation does not influence the trafficking of the α1 subunit [70]. The A322D or D219N mutation was introduced to the α1 subunit using QuikChange II site-directed mutagenesis Kit (Agilent Genomics). DNA sequencing was used to confirm the cDNA sequences. N-terminal HA-tagged full length pCGN-ATF6α (human) plasmid came from Addgene (#11974). The pcDNA3.1-BiP plasmid was provided by Professor Tohru Mizushima (Kumamoto University). The pEGFP-N1 plasmid was a kind gift from Dr. Fraser Moss (Case Western Reserve University). The XBP1s plasmid was a kind gift from Dr Richard N. Sifers. The mouse monoclonal anti-α1 (clone BD24) antibodies came from Millipore (#MAB339). The mouse monoclonal anti-β-actin antibody (#A1978) and anti-FLAG M2 peroxidase antibody (#A8592) were obtained from Sigma. The rabbit polyclonal anti-BiP antibody is from Abgent (#AP50016). The mouse monoclonal ATF6 antibody (#73–505) was from BioAcademia. The rabbit monoclonal anti-Na, K-ATPase was from Abcam (#ab76020). The rabbit polyclonal anti-Matrin-3 antibody is from Bethyl Laboratories (#A300-591A). The mouse monoclonal anti-HA antibody (F-7) is from Santa Cruz (#SC-7392). The rabbit polyclonal anti-XBP1 antibody (M-186) is from Santa Cruz (#SC-7160).

### Cell culture and transfection

HEK293T cells and SH-SY5Y cells were obtained from ATCC. Cells were maintained at 37°C in 5% CO_2_ in Dulbecco’s Modified Eagle Medium (DMEM) (Corning Media) with 10% heat-inactivated fetal bovine serum (Sigma-Aldrich) and 1% Penicillin-Streptomycin (Hyclone). Monolayers were passaged with Trypsin 0.05% (Hyclone). Cells were grown in 6-well plates or 10-cm dishes and allowed to reach ~60–80% confluency before transient transfection using TransIT-2020 (Mirus) according to the manufacturer’s instruction. Cell lines that stably expressing α1β2γ2 and α1(A322D)β2γ2 receptors were generated by transient transfection with α1:β2:γ2 (1:1:1) and α1(A322D):β2:γ2 (1:1:1) plasmids. Then cells were selected using 0.8 mg/mL G-418 (Enzo Life Sciences) and maintained in 0.5 mg/mL G-418. The ATF6 plasmid was transiently transfected at 1 μg/well in 6-well plates, 1.5 μg in 3-cm dishes, or 2 μg in 10-cm dishes using a 1:3 (μg plasmid: μl transfection reagent) ratio. Forty-eight hours post transfection, cells were collected.

To generate a monoclonal HEK293T cells stably expressing α1(A322D)β2γ2 receptors, the α1(A322D) sequence was subcloned into a pIRES2-EGFP bicistronic vector (Clontech) using EcoRI and SacII restriction sites, which would allow the simultaneous expression of α1 subunits and EGFP separately but from the same RNA transcript. Briefly, 6.5 μg of the pIRES2-EGFP vector or 10 μg of the pCMV6-α1 plasmid was added to 4 μl of 10x NEB4 buffer (NEB #B7004S), 1 μl of EcoRI (NEB # R0101S) and 1μl of SacII (NEB #R0157S). 14 μl of RNase-free H_2_O was used to make up to a 40 μl of final volume. The reaction mixture was incubated at 37°C for 4.5 hrs. The reaction samples were then subject to 1% agarose gels to confirm the success of the plasmid digestion. The WT α1 subunit insert (1453 bp) and the digested pIRES2-EGFP vector (5285 bp) were then cut and extracted from the agarose gels using Qiagen Gel Extraction Kit (Qiagen #28704) following the protocol included. 70 ng of the digested pIRES2-EGFP vector and 235 ng of the WT α1 subunit insert (molar ratio of the digested vector and the insert was 1:10) were added with 1 μl of T4 ligase (NEB #M0202S) and 3 μl of T4 ligase buffer (NEB #B0202S), and RNase-free H_2_O was used to make up to a 30 μl final volume. The reaction mixture was incubated at room temperature for 3 hrs and transformed into the DH5α competent cells (Invitrogen). The resulting pIRES2-EGFP-α1 plasmid was confirmed by DNA sequencing. The A322D mutation in the α1 subunit was generated using QuikChange II site-directed mutagenesis Kit (Agilent Genomics). After transfection and G-418 treatment, cells that were GFP-positive were considered as those successfully transfected with the α1(A322D) subunit. GFP-positive cells were further diluted into 96-well plates, allowing a single cell distribution in each well. Cells with robust GFP signals were further selected to grow to population.

### Western blot analysis

Cells were harvested with Trypsin-EDTA (0.05%) (Hyclone). Cells were lysed in lysis buffer (50 mM Tris, pH 7.5, 150 mM NaCl, and 1% Triton X-100) supplemented with Roche complete protease inhibitor cocktail on ice for an hour and then subject to centrifugation (13,400 × *g*, 15 min, 4°C) to remove cell debris and nucleus. The supernatant was collected as the total cellular protein. Protein concentration was measured using MicroBCA assay (Pierce). Endoglycosidase H (endo H) digestion and Peptide-N-Glycosidase F (PNGase F) (New England Biolabs) digestion were performed according to the published procedure [71]. Loading samples were generated by mixing cell lysates and 4x SDS sample loading buffer (Biorad) and separated in an 8% denaturing tris-glycine gel. Western blot analysis was performed using corresponding antibodies. Band intensity was quantified using Image J software from the NIH. The average normalized band intensity to the protein gel loading control was plotted.

### Resazurin cell toxicity assay

HEK293T cells stably expressing α1(A322D)β2γ2 receptors were plated into a 96-well plate. The cells were separated into 7 groups which were treated with DMSO, BIX (1.2 μM, 6 μM, 12 μM, 24 μM, or 48 μM) for 24h, or thapsigargin (2 μM, 7h). Resazurin (0.15 mg / ml dissolved in DPBS) was added to cells for 1.5 h before plate reading. Fluorescence signal at 560 nm excitation / 590 nm emission was measured.

### Nuclear extraction

The nuclear extraction was performed according to published procedure [72]. Cells were harvested with Trypsin-EDTA (0.05%) (Hyclone) and lysed on ice for 5 min with harvest buffer (10 mM HEPES pH 7.9, 50 mM NaCl, 0.5 M Sucrose, 0.1 mM EDTA, 0.5% Triton X-100) supplemented with 1 mM DTT and the Roche complete protease inhibitor cocktail. The lysates were then centrifuged at 1000 g for 10 mins at 4°C. The supernatant was collected as the cytoplasmic part. The pellet was re-suspended with buffer A (10 mM HEPES pH 7.9, 10 mM KCl, 0.1 mM EDTA, 0.1 mM EGTA) supplemented with 1 mM DTT and the Roche complete protease inhibitor cocktail and then subjected to another centrifugation at 1000 g for 5 mins at 4°C. The resulting pellet was re-suspended with buffer C (10 mM HEPES pH 7.9, 500 mM NaCl, 0.1 mM EDTA, 0.1 mM EGTA, 0.1% IGEPAL (NP40)) supplemented with 1 mM DTT and the Roche complete protease inhibitor cocktail, incubated on ice for 1 h, and then subject to centrifugation at 16000 g for 15 min at 4°C. The resulting supernatant was collected as the nuclear extract of the cells.

### Biotinylation of cell surface proteins

HEK293T cells transfected with GABA_A_ receptors were plated in 10-cm dishes for surface biotinylation experiments according to published procedure [54]. In brief, cells were either transfected with GFP and ATF6 plasmids for 48 hrs or treated with BIX at 12 μM for 24 hrs. Then, intact cells were rinsed gently twice with ice-cold PBS and incubated with the membrane-impermeable biotinylation reagent Sulfo-NHS SS-Biotin (0.5 mg ⁄ mL; Pierce) in PBS for 30 min at 4°C to label surface membrane proteins. Glycine (10 mM) in ice-cold PBS was added to cells for 5 min at 4°C to quench the reaction. N-ethylmaleimide (NEM, 5 nM) in PBS was added for 15 min at room temperature to block the Sulfhydryl groups. Cells were then solubilized for 1 h at 4°C in lysis buffer (Triton X-100, 1%; Tris–HCl, 50 mM; NaCl, 150 mM; and EDTA, 5 mM; pH 7.5) supplemented with Roche complete protease inhibitor cocktail and 5 mM NEM. The lysates were centrifuged (16,000 × g, 15 min at 4°C), and the supernatant contained the biotinylated surface proteins. The concentration of the supernatant was measured using MicroBCA assay (Pierce). Biotinylated surface proteins were purified by incubating the above supernatant for 1 h at 4°C with 30 μL of immobilized neutravidin-conjugated agarose bead slurry (Pierce), and being subjected to centrifugation (16000 ×g, 10 mins). The beads were washed three times with buffer (Triton X-100, 0.5%; Tris–HCl, 50 mM; NaCl, 150 mM; and EDTA, 5 mM; pH 7.5). Surface proteins were eluted from beads by boiling for 5 mins with 60 μL of LSB ⁄ Urea buffer (2x Laemmli sample buffer (LSB) with 100 mM DTT and 6 M urea; pH 6.8) before subjected to SDS-PAGE and Western blotting analysis.

### Cycloheximide-chase assay

Single clone stable α1(A322D)β2γ2 GABA_A_ receptors HEK293T cells were plated in to 6-well plates one or two days before transfection. Cells then transfected with GFP or treated with DMSO as control group and transfected with ATF6 or XBP1s or treated with BIX 12 μM 24 hr using transfection protocol listed above. Cells were treated with 150 μg/mL cycloheximide (Ameresco) to stop protein translation for the 2 hrs, 1 hr, and 0.5 hr before being collected for Western blot analysis.

### Whole-cell patch clamp electrophysiology recording

The whole-cell patch clamp procedure follows the protocol described in detail before [55]. In brief, whole-cell currents were recorded 24 hrs post application of BIX (12μM) in monoclonal HEK293T cells stably expressing α1(A322D)β2γ2 receptors. The electrode internal solution contains 153 mM KCl, 1 mM MgCl_2_, 5 mM EGTA, 10 mM HEPES, and 2 mM MgATP (pH 7.3), whereas the external recording solution contains 142 mM NaCl, 8 mM KCl, 6 mM MgCl_2_, 1 mM CaCl_2_, 10 mM glucose, 10 mM HEPES, and 120 nM Fenvalerate (pH 7.4). Coverslips containing HEK293T cells were placed in a RC-25 recording chamber (Warner Instruments) on the stage of an Olympus IX-71 inverted fluorescence microscope. Cells were perfused with external solution. A Quartz MicroManifold with 100-μm inner diameter inlet tubes (ALA Scientific) was placed so that its tip was within 50 μm of the cell to be recorded. The whole-cell GABA-induced currents were recorded at a holding potential of -60 mV in voltage clamp mode using an Axopatch 200B amplifier (Molecular Devices). The pClamp10 software was used for data acquisition and analysis.

### Statistical analysis

All data were presented as mean ± SEM. Statistical significance was evaluated using two-tailed Student’s t-Test if two groups were compared and one-way ANOVA followed by post-hoc Tukey or Fisher test if more than two groups were compared. A *p* value of less than 0.05 was considered statistically significant.

## Supporting information

S1 Fig Influence of GABA_A_ receptor protein expression levels on the effect of BIX treatment. **(A**) HEK293T cells were either transiently transfected with 0.15 μg α1(A322D) subunit, 0.15 μg β2 subunit, and 0.15 μg γ2 subunit of GABA_A_ receptors or 0.25 μg α1(A322D) subunit, 0.25 μg β2 subunit, and 0.25 μg γ2 subunit of GABA_A_ receptors. 0.15 μg per subunit group and 0.25 μg per subunit group were then treated with BIX (12 μM, 24h) or DMSO as controls. Forty-eight hours post transfection, cells were lysed, and total proteins were extracted. The cell lysates were then subjected to SDS-PAGE and Western blot analysis using corresponding antibodies. Quantification of total cellular protein expression levels of α1 and BiP is shown in **B** & **C** (n = 4, paired t-test). *, p<0.05.
(PDF)
