# Supplementary figures and images for: Remodeling the endoplasmic reticulum proteostasis network restores proteostasis of pathogenic GABAA receptors
Source: PLoS One. 2018 Nov 27;13(11):e0207948. doi: 10.1371/journal.pone.0207948 (PMC6258528; doi:10.1371/journal.pone.0207948)

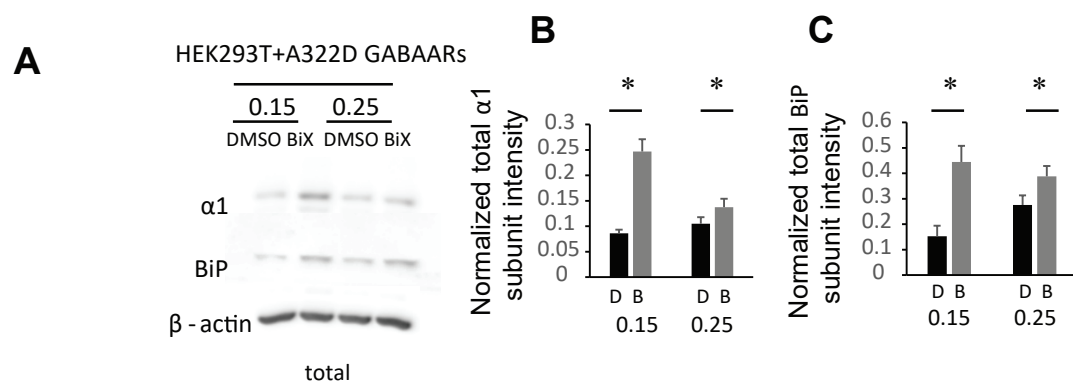

Supplementary Figure 1

Supplement: S1 Fig — (A) HEK293T cells were either transiently transfected with 0.15 μg α1(A322D) subunit, 0.15 μg β2 subunit, and 0.15 μg γ2 subunit of GABAA receptors or 0.25 μg α1(A322D) subunit, 0.25 μg β2 subunit, and 0.25 μg γ2 subunit of GABAA receptors. 0.15 μg per subunit group and 0.25 μg per subunit group were then treated with BIX (12 μM, 24h) or DMSO as controls. Forty-eight hours post transfection, cells were lysed, and total proteins were extracted. The cell lysates were then subjected to SDS-PAGE and Western blot analysis using corresponding antibodies. Quantification of total cellular protein expression levels of α1 and BiP is shown in B & C (n = 4, paired t-test). *, p<0.05. (PDF) [file pone.0207948.s001.pdf]
